# Having a Coffee Break: The Impact of Caffeine Consumption on Microglia-Mediated Inflammation in Neurodegenerative Diseases

**DOI:** 10.1155/2017/4761081

**Published:** 2017-01-31

**Authors:** Maria H. Madeira, Raquel Boia, António F. Ambrósio, Ana R. Santiago

**Affiliations:** ^1^Institute for Biomedical Imaging and Life Sciences (IBILI), Faculty of Medicine, University of Coimbra, 3000-548 Coimbra, Portugal; ^2^CNC.IBILI Consortium, University of Coimbra, 3004-504 Coimbra, Portugal; ^3^Association for Innovation and Biomedical Research on Light and Image (AIBILI), 3000-548 Coimbra, Portugal

## Abstract

Caffeine is the major component of coffee and the most consumed psychostimulant in the world and at nontoxic doses acts as a nonselective adenosine receptor antagonist. Epidemiological evidence suggests that caffeine consumption reduces the risk of several neurological and neurodegenerative diseases. However, despite the beneficial effects of caffeine consumption in human health and behaviour, the mechanisms by which it impacts the pathophysiology of neurodegenerative diseases still remain to be clarified. A promising hypothesis is that caffeine controls microglia-mediated neuroinflammatory response associated with the majority of neurodegenerative conditions. Accordingly, it has been already described that the modulation of adenosine receptors, namely, the A_2A_ receptor, affords neuroprotection through the control of microglia reactivity and neuroinflammation. In this review, we will summarize the main effects of caffeine in the modulation of neuroinflammation in neurodegenerative diseases.

## 1. Microglial Cells Play Crucial Roles in Neurodegenerative Diseases

The multitaskers microglial cells are active effectors and regulators of homeostasis in the central nervous system (CNS). Microglial cells constantly survey the surrounding environment, and as primary resident immune cells in the CNS, they respond to the presence of pathogens, stress, or injury [1]. In fact, for decades, it was believed that in homeostatic conditions microglial cells were in a nonreactive* resting *state, which could be transformed into a* reactive *state under pathological conditions. Nevertheless, the crucial role of microglial cells in the noninjured CNS has become more evident in recent years, and these cells not only are involved in immune pathological response but are essential during CNS development, participating in crucial processes such as in synaptic pruning [2, 3] and synaptic plasticity [4, 5]. Thus, the so-called* resting phenotype* should also reflect an active state and should be replaced by* surveillance state *[6].

Contrasting to the highly ramified organization presented by microglia in the* surveillance state*, reactive microglial cells are characterized by amoeboid morphology, which favours phagocytosis [7, 8]. This shift to a more activated phenotype results in increased release of proinflammatory and cytotoxic factors, such as tumour necrosis factor (TNF), interleukin-1*β* (IL-1*β*), nitric oxide (NO), and reactive oxygen species (ROS) [9], as well as in increased expression of surface molecules related to the innate immune response, as major histocompatibility complex (MHC) proteins and antigen-presenting receptors [10].

When studying microglial cell reactivity in the context of pathology, one major point is the dichotomy between their contribution to neuroprotection and neurodegeneration. Microglia activation and production of inflammatory mediators are known to be a response to neuronal dysfunction and death to control the damage and to promote recovery (reviewed in [11, 12]). Nevertheless, sustained reactivity of microglial cells has a detrimental role and contributes to neurodegeneration, in which neuronal loss is accompanied by increased neuroinflammatory conditions [13–16].

In the two last decades, significant advances have been made in the understanding of the contribution of microglial cells to CNS diseases. The activation of these cells is recognized as a hallmark of a wide variety of neurodegenerative diseases, such as Alzheimer's disease (AD), Parkinson's disease (PD), and multiple sclerosis (MS), and in retinal diseases, such glaucoma, diabetic retinopathy, and age-related macular degeneration (reviewed in [9, 17]). Hence, suppression of microglial-associated deleterious effects has emerged as a potential therapeutic strategy to prevent neurodegeneration [18, 19].

## 2. The Modulation of the Adenosinergic System for Therapeutic Intervention in Neurodegenerative Diseases 

Adenosine is a ubiquitously expressed purine nucleoside that acts as a homeostatic factor and a crucial neuromodulator in the CNS. In physiological conditions, the concentration of adenosine in the extracellular fluids is low (30–300 nM), but its levels increase to 10 *μ*M or higher during enhanced nerve activity, hypoxia, ischemia, or CNS damage [20]. At high concentrations, adenosine is able to modulate the release of excitotoxic mediators, limit calcium influx, hyperpolarize neurons, and exert modulatory effects on glial cells [21].

Four types of G-coupled receptors coordinate cellular responses to extracellular adenosine: the inhibitory A_1_ and A_3_ receptors and the facilitatory A_2A_ and A_2B_ receptors [22]. These receptors are expressed on astrocytes, microglia, and infiltrating immune cells and regulate the immune response of these cells in the CNS [23–31]. The actions mediated by adenosine in the immune cells may be towards neuronal protection, but adenosine may also promote proinflammatory response, leading to neuronal damage (reviewed [32]).

In the last decades, the neuroprotective properties of adenosine in the CNS have been extensively documented [33–41]. The neuromodulatory effects mediated by adenosine rely on a balanced activation of the inhibitory A_1_ receptor (A_1_R) and excitatory A_2A_ receptor (A_2A_R) [41]. A large body of evidence points to a neuroprotective role of A_1_R activation, but this receptor is prone to rapid desensitization, limiting the time-lapse of action of possible neuroprotective therapies [41].

Concerning A_2A_R, there is an apparent paradox on the role mediated by this receptor in inflammation. In the periphery, activation of A_2A_R signaling suppresses inflammation [42], attenuates pulmonary ischemic injury [43, 44], and improves cardiac dysfunction [45]. In the CNS, A_2A_R controls the release of BDNF from activated microglia [46], and its blockade prevents hippocampal LPS-induced neuroinflammation [47] and prevents IL-1*β*-induced exacerbation of neuronal toxicity [48]. Antagonists A_2A_R prevent retinal microglia reactivity, affording protection to retinal neuronal cells [26, 27]. Importantly, blockade of A_2A_R has been shown to confer neuroprotection against a broad spectrum of CNS insults [49, 50].

While in the periphery, the activation of A_2A_R halts a rapid immune response (acute), in the CNS the activation of A_2A_R aggravates the inflammatory response (chronic conditions) (reviewed in [51]). This dual role of A_2A_R might reflect the complexity of actions in distinct cell types present in the CNS, which may lead to distinct effects upon CNS injury [41, 52]. The mechanisms by which the blockade of A_2A_R is able to impact neuroprotection remains to be clarified, but two leading hypotheses have been explored: the control of glutamate excitotoxicity and the control of microglia-mediated neuroinflammation [40, 41].

The blockade of A_2A_R has emerged as a potential therapeutic strategy, based on its ability to regulate proliferation, chemotaxis, and reactivity of glial cells, affording protection in several brain diseases (reviewed in [40, 50]).

Although less expressed, A_2B_R and A_3_R may also mediate protective effects in the CNS. By acting on A_2B_R, adenosine has been shown to augment the production of IL-10 by microglial and macrophages cells, while preventing the release of proinflammatory cytokines [53]. The activation of A_3_R has been shown to afford protective effects, namely, after brain ischemia [54] and in retinal neurodegeneration [55].

## 3. Caffeine: An Antagonist of Adenosine Receptors with Protective Functions in the CNS

Caffeine (1,3,7-trimethylxanthine) is the most widely consumed psychostimulant substance in the world, mainly found in dietary sources, such coffee, tea, and energy drinks [56]. Caffeine has been described as a CNS stimulant that promotes wakefulness, enhances mood and cognition, and produces stimulatory effects [57, 58]. In fact, caffeine exerts beneficial effects on human behaviour, which were not mimicked by the consumption of decaffeinated drinks [59].

Worldwide, it is estimated that caffeine consumption, from all sources, is around 70 to 76 mg/person/day. A single cup of coffee provides a dose of 0.4 to 2.5 mg/kg of caffeine, leading to a peak serum concentration of 0.25 to 2 mg/L or approximately 1 to 10 *μ*M. In humans, 99% of caffeine is absorbed from the gastrointestinal tract in about 45 min after ingestion [56]. The first metabolic step, which represents on average 80% of the total process, is via N-3 demethylation to paraxanthine (1,7-dimethylxanthine) by the cytochrome P450 1A2 enzyme [60] and was recently found to be associated with the variability of caffeine consumption between individuals [61]. Other two important products of caffeine metabolism are theobromine (3,7-dimethylxanthine) and theophylline (1,3-dimethylxanthine), which represent about 16% of the total metabolites [62]. After long-term consumption of high doses of caffeine, these metabolites can also contribute to its pharmacological actions, since it can result in an accumulation of methylxanthines in the body, due to end-product inhibition of demethylation, and thereby should be also considered when investigating the pharmacological actions of caffeine [63].

Most of the studies about the beneficial effects of coffee have been focused largely on caffeine, but coffee contains over 1,000 components that may have neuroprotective effects [64–67]. Interestingly, decaffeinated coffee is protective in* Drosophila* models of PD [68], suggesting that other coffee constituents may provide neuroprotection. Eicosanoyl-5-hydroxytryptamide, a constituent of coffee, has been demonstrated to ameliorate the phenotype of a PD model associated with decreased protein aggregation and phosphorylation, improved neuronal integrity, and reduced neuroinflammation [69]. Also, chlorogenic acid, trigonelline, and melanoidins are also able to impact gene transcription and regulation of body fat percentage [70, 71].

The biochemical mechanisms that underlie the actions of caffeine are dependent on the dose. In the brain, the molecular targets of caffeine at nontoxic doses are the adenosine receptors A_1_ and A_2A_ [56].

One of the most recognized actions of caffeine is its ability to reduce sleep and sleepiness. Caffeine, acting on A_2A_R, promotes wakefulness, as demonstrated by genetic manipulation of the A_2A_R in the nucleus accumbens [72].

Ethanol and caffeinated beverages are frequently consumed in combination, a fact that might be due to the popular belief that caffeine can offset the acute intoxicating actions of ethanol. In fact, it has been shown that caffeine is able to attenuate ethanol-induced motor incoordination in rats [73], an effect that was also observed with A_1_R antagonists, but not with antagonists of A_2A_R. Interestingly, caffeine administration also prevents the hypnotic effects induced by ethanol, an effect suggested to be mediated by A_2A_R antagonism, since knockout (KO) mice for this receptor display similar behaviour [74].

Caffeine is also associated with alterations in neurotransmitter release and increase neuronal firing (via A_1_R), as well as enhancing dopaminergic transmission (via A_2A_R), globally affecting neuronal processes associated with mood and cognition (reviewed in [56]). Caffeine has been shown to control synaptic plasticity [75], to revert memory impairments [76, 77], and to prevent mood alteration triggered by chronic stress [78]. Importantly, these effects were also observed in the presence of selective A_2A_R antagonists prompting the critical role of this receptor to the actions of caffeine. Indeed, using A_2A_R-KO mice, it was recently shown that the neuroprotective effects of caffeine in a PD model rely on the presence of A_2A_R [79].

Several studies have been demonstrating protective effects of caffeine in patients and animal models of neurodegenerative diseases, mainly by reducing excitotoxicity, apoptosis, and neuroinflammation (reviewed in [80]).

## 4. Modulation of Microglia Reactivity and Neuroinflammation with Caffeine

Since the late 1990s, several studies have shown that caffeine reduces neuroinflammation in models of AD and PD (reviewed in [80]). Additionally, epidemiological studies have shown that caffeine might exert neuroprotective effects in humans [56, 81, 82].

Several studies have also focused their attention on the ability of caffeine to reduce microglia reactivity. In an* in vitro* system, using the murine BV-2 microglia cell line, it was demonstrated that 2 mM caffeine attenuates the expression of proinflammatory mediators, such as NO and TNF, and their regulatory genes, elicited by lipopolysaccharide (LPS) [83], widely known to induce potent neuroinflammatory responses in the brain [84]. The same study suggests the modulation of extracellular signal-regulated kinase (ERK) signaling cascade and consequent NF-*κ*B activation as a main pathway for caffeine actions [83], which has been also related to the A_2A_R activation-induced macroglial cell reactivity [85]. In an animal model of inflammation, in which LPS was infused over a period of two or four weeks in the brain, caffeine administration (daily intraperitoneal injection) reduces LPS-induced microglia activation in three regions of the hippocampus, in a dose-dependent manner [86].

Importantly, caffeine alters neuronal functioning in physiological brain conditions, increasing the spontaneous firing (reviewed in [56]). The effects of caffeine in nonneuronal cells in nonpathological conditions have not been extensively studied. It was already described that brain sections of animals administered with caffeine* ad libitum *in the drinking water have altered microglia density and morphology, as observed by process retraction and enlargement of the cell soma, indicating a more reactive phenotype [87]. The authors suggested that caffeine might prime microglial cells, impacting the transition from the surveillance to the reactive state [88]. Notably, in the retina, caffeine intake does not change microglia reactivity and expression of proinflammatory markers [87].

## 5. Beneficial Effects of Caffeine in Alzheimer's Disease: Neuroinflammation and Neuroprotection

Alzheimer's disease is the most common type of dementia worldwide, clinically characterized by a progressive decline of cognitive functions and memory deficits [89]. The main neuropathological hallmarks of AD are extraneuronal deposition of amyloid-beta (A*β*) protein in the form of plaques and intraneuronal aggregation of the hyperphosphorylated microtubule-associated protein tau in the form of filaments, mainly in the cortex, hippocampus, and amygdala [90]. Furthermore, a strong neuroinflammatory component has been associated with AD pathology, with increased glial cell reactivity (microgliosis and astrogliosis), activation of both classic and alternate pathways of the complement system, upregulation of inflammatory markers, and increased phagocytic activity [91, 92].

The presence of A*β* oligomers has been described to lead to microglia-mediated neuroinflammatory response, with alterations in the phagocytic efficiency and sustained overproduction of inflammatory mediators, which may contribute to neurotoxicity and neuronal loss [93]. Indeed, microglia reactivity has been described not only in the brain, but also in the retinas of AD animal models [94] and patients [95]. It remains to be elucidated whether microglia activation is a cause or a consequence of AD, but the role of microglia reactivity in the progression of the disease is unquestionable. Hence, interventions targeted to control microglial cell reactivity might delay the progression of AD.

Consumption of caffeine has been associated with reduction in the cognitive decline in healthy subjects (with advanced age) and also AD patients [96–99]. In AD animal models the beneficial effects of caffeine intake include amelioration of cognitive impairments [100, 101] and dementia [102].

It has been described that increased caffeine levels in the plasma are associated with reduced inflammatory cytokine levels in the hippocampus [103]. Remarkably, chronic administration of caffeine to a transgenic mouse model of progressive AD-like tau pathology mitigates several proinflammatory and oxidative stress markers in the hippocampus and prevents the development of spatial memory deficits [104].

Disruption of the blood-brain barrier (BBB) is an early pathological event in AD [105, 106] and may potentiate the accumulation of A*β* in the brain by allowing the transport of A*β* produced in the periphery [107]. Caffeine administration protects against AD-associated BBB dysfunction [106, 108] and reduces glial cell reactivity at sites of BBB leakage [106]. The effects of caffeine in the control of BBB integrity have been associated with its antagonistic actions on adenosine receptors and consequent inhibition of cyclic adenosine monophosphate (cAMP) activity and control of calcium intracellular stores [108]. Caffeine might control AD-associated increase on inflammatory mediators by reducing glial cell reactivity on the BBB leakage site [106] through a reduction in infiltration of immune cells from the periphery [109].

Moreover, using an animal model showing age-related CNS alterations that includes cognitive impairment, increased neuroinflammatory markers, and neurodegeneration, chronic administration of caffeine improves memory deficits and reduces the expression of ROS and proinflammatory cytokines TNF and IL-1*β*, further conferring antiapoptotic effects [110]. Similarly, the effects of caffeine are mimicked by selective antagonists of A_2A_R [111], suggesting that the actions of caffeine are mediated by the blockade of A_2A_R. In accordance, both pharmacological blockade and genetic inactivation of A_2A_R afford neuroprotection against A*β* toxicity [112].

These reports reinforce the crucial importance of A_2A_R in A*β* toxicity and in the associated microglia reactivity and neuroinflammatory response in the context of AD, demonstrating also prophylactic properties of caffeine and the therapeutic potential of A_2A_R antagonists for the treatment of AD [113].

## 6. Caffeine Modulates Neuroinflammation in Parkinson's Disease: Possible Strategy for Neuroprotection?

Parkinson's disease (PD) is the second most common progressive neurodegenerative disorder. It is characterized by a progressive loss of dopaminergic neurons of the nigrostriatal pathway with the occurrence of Lewy bodies (abnormal deposits of *α*-synuclein), which clinically translates in muscular rigidity, resting tremor, bradykinesia, and postural instability [114]. The pathogenesis of PD has been also associated with chronic neuroinflammation [115] and oxidative stress [116], both contributing to BBB disruption [116, 117].

The brain is particularly susceptible to oxidative stress due to the high consumption of oxygen [116]. Oxidative stress has been associated with several neurodegenerative diseases, including PD. Indeed, there is evidence from postmortem human samples that oxidative stress might be a primary insult that leads to neuronal damage in PD [118, 119]. In substantia nigra, microglial cells have been proposed to be the main cells producing oxidative stress products [120], suggesting the involvement of these cells in the pathophysiology of PD. The involvement of neuroinflammation in PD was suggested after observation of increased number of reactive microglial cells and an upregulation of major histocompatibility complex class II (MHC-II) in PD patients [121]. Indeed, reactive microglia and neuroinflammatory response have been strongly associated with dopaminergic cell loss in PD (reviewed in [122–124]). Furthermore, elevated levels of proinflammatory cytokines such as TNF [125], IL-1*β*, and IL-6 [126] have been described in the striatum of PD patients. In the 1-methyl-4-phenyl-1,2,3,6-tetrahydropyridine (MPTP) animal model of PD there is evidence demonstrating the neurotoxic contribution of microglia-produced TNF [127], IL-1*β* [128], IL-6, and NO [129] to the loss of dopaminergic neurons. These proinflammatory cytokines, along with factors released from the dying dopaminergic cells, seem to increase and sustain neuroinflammation, leading to an irreversible loss of dopaminergic neurons (reviewed in [130]). Hence, future therapeutic strategies should consider inhibition of microglia-mediated neuroinflammation, possibly in combination with neurotropic factors, aiming to delay the progression of PD.

Epidemiological studies have been associating the consumption of caffeine with reduced risk of developing PD [131–134]. Using the MPTP mouse model, it was shown that daily intraperitoneal administration of caffeine attenuates microglia reactivity and prevents BBB dysregulation, leading to decreased dopaminergic neuronal loss [135, 136]. Accordingly, even when introduced in the later phases of the neurodegenerative process, caffeine is also able to attenuate the inflammatory process and microglial cell expression of CD68 (a marker of reactive microglia), which suggests its ability to arrest or delay neuroinflammation and neurodegeneration [135]. Likewise, caffeine, even in low doses, is able to reverse functional motor deficits in PD animal models [137, 138].

Although the mechanisms underlying neuroprotection by caffeine remain a matter of debate, it has been widely suggested that the neuroprotective effects of caffeine involve the antagonism of A_2A_R [79, 139, 140]. Notably, pharmacological blockade of A_2A_R presents similar protective effects to the ones observed with caffeine in several experimental models of PD [35, 140–142]. Indeed, the critical contribution of A_2A_R to caffeine-mediated neuroprotection was recently demonstrated in mice lacking the A_2A_R gene (KO mice) and exposed to MPTP. In these animals, caffeine had no effect on MPTP toxicity, namely, in striatal neuronal loss and motor activity impairment [79].

The selective A_2A_R antagonists istradefylline (KW 6002) [143] and preladenant (SCH 420814) [144] have been investigated in the past years in clinical trials for PD. The A_2A_R antagonists significant ameliorate the motor symptoms, but more studies are required to establish the clinical utility of these drugs [143, 144].

Taking into account the contribution of microglia-mediated neuroinflammation in the pathophysiology of PD and the beneficial effects of caffeine and A_2A_R antagonists, one can hypothesize that pharmacologic blockade of A_2A_R might offer potential therapeutic benefit in PD at the level of motor alterations, neuroinflammatory response, and neuroprotection.

## 7. The Effects of Caffeine in Multiple Sclerosis

Multiple sclerosis (MS) is an autoimmune, inflammatory disease of the CNS and the most common cause of chronic neurologic disability beginning in early to middle adult life [145]. The major pathological hallmarks of MS include dysregulation of BBB, which promotes macrophage and lymphocyte infiltration, and the presence of sclerotic plaques in the CNS [146]. In more advanced stages, the degenerative phase is characterized by demyelination and axonal damage that results in neuronal functional impairment in the brain and in the spinal cord. The demyelination process is associated with inflammation, which can occur through activation of resident astrocytes and microglia and by the inflammatory cytokine products of infiltrating immune cells (lymphocytes or macrophages) [147].

Although the exact role of microglial cells in MS is not completely elucidated, it is recognized that these cells are able to sustain and propagate the inflammatory response during autoimmune inflammation [148]. Reactive microglia, expressing MHC-II, exert functions of antigen-presenting cells during MS, therefore promoting the propagation of the inflammatory process and secretion of cytokine or chemokine [149]. Indeed, the detrimental role of microglia activation in MS models has been demonstrated, with the inhibition of these cells leading to a reduction in the myelin and axonal damage, and also in neurodegeneration [148, 150–152].

Nonetheless, microglial cells not only contribute to the neurodegenerative process, but also play an important role in the promotion of neuroprotection, downregulation of inflammatory process, and stimulation of tissue repair. This complex and dual role might be due to the high heterogeneity of myeloid populations (microglia, monocytes, and infiltrating T-cells), with distinct subtypes and distinct states of microglia reactivity (M1 and M2) associated with different pathologic or protective roles [153–155].

As previously mentioned, several reports have implicated the modulation of adenosine receptors in immune cells to a suppression of the inflammatory response (reviewed by [156]). Indeed, the levels of adenosine are reduced in the plasma of MS patients and the expression of A_2A_R and A_1_R is up- and downregulated, respectively [157, 158]. Studies in animal models of MS confirmed the decreased expression of A_1_R in microglial cells and an increase in both pro- and anti-inflammatory mediators [159, 160]. The administration of caffeine to these animals restores the levels of A_1_R and attenuates the neuroinflammatory process and demyelination [160]. In accordance with studies in animal models of MS, high consumption of coffee may decrease the risk of developing MS [65, 161]. The authors suggest the suppression of the neuroinflammatory process and consequent production of proinflammatory cytokines as the mechanism underlying the observed association [65].

Similar to other brain conditions, the levels of A_2A_R have been shown to be upregulated in the brain [162] and in lymphocytes of MS patients [163]. Activation of A_2A_R has been associated with a strong anti-inflammatory response by immune cells [156, 164]. Correspondingly, genetic inactivation of A_2A_R has been reported to enhance the inflammatory cell infiltration and microglial cell activation in cortex, brainstem, and spinal cord in a MS animal model, also increasing demyelination and axonal damage [165]. These results suggest that adenosine acting on A_2A_R triggers neuroprotective effects. Intriguingly, the use of antagonists of A_2A_R also affords neuroprotection in a model of MS by reducing lymphocyte infiltration [166]. Indeed, a dual role for A_2A_R in autoimmune inflammation has been already described, with activation of A_2A_R leading to prevention of the disease in the early stages, whereas A_2A_R blockade affords protection in later stages by reducing neuroinflammation [167]. These results suggest that A_2A_R activity can impact the progression of the disease in multiple cellular and molecular targets, but we must keep in mind the possibility that the genetic deletion and pharmacological inactivation of the receptor produce opposite effects in the pathology. Global genetic deletion of A_2A_R occurs in all cellular elements, whereas the pharmacological blockade is suggested to target preferentially neutrophils and lymphocytes [168], reducing their infiltration and therefore exerting neuroprotective effects, as well as reducing microglia activation [40, 165]. Therefore, the A_2A_R has been considered a potential target for therapeutic approaches in MS. Still, chronic treatment with caffeine during the degenerative phase of MS animal model provides neuroprotection regardless of the A_2A_R genotype, implying that, in this disease, caffeine acts in a non-A_2A_R-dependent manner [169].

## 8. Beneficial Properties of Caffeine beyond Brain Neurodegenerative Diseases: A Look into the Retina

Despite the extensive evidence regarding the effects of caffeine consumption in the brain, very little is known about the effects of caffeine consumption in retinal degeneration [170]. We have shown that caffeine administration reduces retinal neuroinflammation and microglial reactivity in an animal model of retinal degeneration induced by ischemia reperfusion (I-R). Notably, caffeine treatment is also able to prevent retinal neuronal cell apoptosis in these animals [171]. Accordingly, in animals subjected to I-R, pharmacological blockade of A_2A_R prevents microglia reactivity and neuroinflammatory response [26]. Using retinal organotypic cultures and an I-R animal model we demonstrated that blockade of A_2A_R confers neuroprotection in the retinal through the control of microglia-mediated neuroinflammation [26, 87]. Hence, taking into account the antagonistic effects of caffeine in the A_2A_R, one can hypothesize that protection against neuronal apoptosis in the retina afforded by caffeine might also be due to a reduction in microglia reactivity and neuroinflammatory response.

In addition, very recently, we demonstrated that caffeine intake prevents microglia-mediated neuroinflammation and increases the survival of retinal ganglion cells in an animal model of glaucoma [87], suggesting that caffeine may have a prophylactic effect in glaucoma. Still, the understanding of the effects of caffeine and A_2A_R antagonists in retinal neuroinflammation and neurodegeneration is still in a very early stage, but it appears as a promising therapeutic strategy for retinal neurodegenerative diseases [170].

## 9. Conclusions

Coffee is one of the most consumed beverages worldwide and its consumption has been demonstrated to impact human health. Taking in account the beneficial properties of caffeine in neurological and neurodegenerative diseases and the molecular targets of caffeine in the CNS, it is very important to elucidate the effects of caffeine to neuroinflammation.

**Figure 1:**
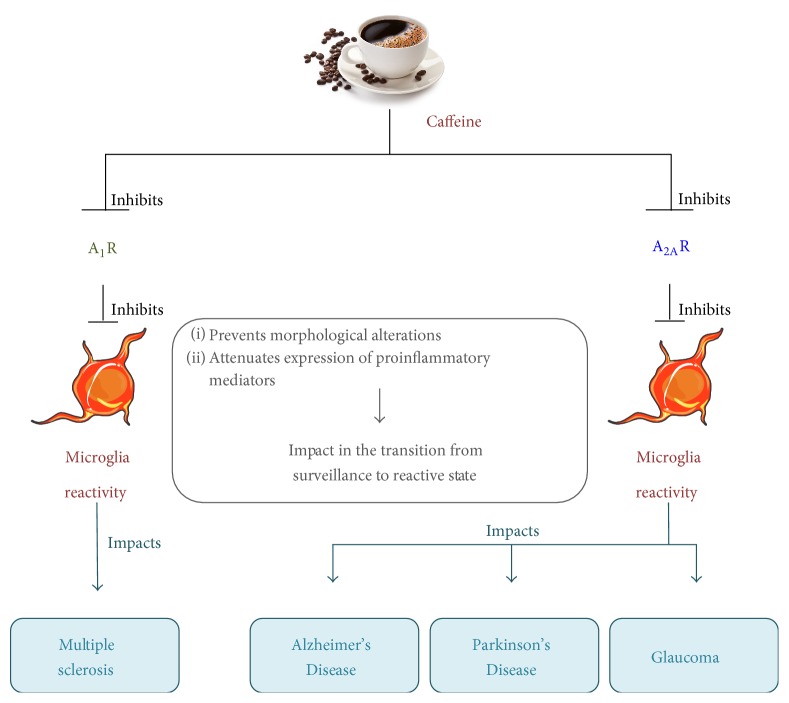
Caffeine reduces microglia-mediated inflammatory environment on CNS degenerative diseases. Schematic summary of the effects of caffeine intake on microglia reactivity and the associated CNS degenerative diseases.

Antagonists of adenosine receptors, namely, of A_2A_R, have been vastly studied in neurodegenerative diseases. One hypothesis that has been gaining attention to explain the protective properties of caffeine and A_2A_R antagonists is the control of microglia-mediated neuroinflammation (Figure 1). Caffeine may block A_2A_R in microglial cells thus controlling exacerbated microglia reactivity and noxious inflammation, providing neuroprotection. Nevertheless, more studies are required to elucidate the cellular and molecular mechanisms of caffeine and its metabolites in the modulation of microglia-mediated neuroinflammation in neurodegenerative disorders.
